# Electrocardiographic Alterations in Obstructive Sleep Apnea Syndrome: Mechanisms and Clinical Relevance

**DOI:** 10.3390/life16020251

**Published:** 2026-02-02

**Authors:** Andrea Segreti, Michele Pelullo, Virginia Ligorio, Aurora Ferro, Riccardo Cricco, Martina Ciancio, Simone Pasquale Crispino, Francesco Grigioni

**Affiliations:** 1 Cardiology Unit, Fondazione Policlinico Universitario Campus Bio-Medico, Via Alvaro del Portillo, 200, 00128 Rome, Italy; michele.pelullo@unicampus.it (M.P.); virginia.ligorio@unicampus.it (V.L.); riccardo.cricco@unicampus.it (R.C.); martina.ciancio@unicampus.it (M.C.); simone.crispino@policlinicocampus.it (S.P.C.); f.grigioni@policlinicocampus.it (F.G.); 2 Research Unit of Cardiovascular Science, Department of Medicine and Surgery, Università Campus Bio-Medico di Roma, Via Alvaro del Portillo, 21, 00128 Rome, Italy

**Keywords:** obstructive sleep apnea, electrocardiogram, atrial arrhythmias, ventricular arrhythmias, artificial intelligence

## Abstract

Obstructive Sleep Apnea (OSA) is a highly prevalent yet frequently underdiagnosed disorder strongly associated with cardiovascular morbidity and mortality. It is characterized by recurrent episodes of intermittent hypoxia, intrathoracic pressure swings, and sleep fragmentation that trigger sympathetic hyperactivation, oxidative stress, systemic inflammation, and progressive structural cardiac remodeling. These mechanisms translate into a wide range of electrocardiographic (ECG) abnormalities, including both nocturnal brady- and tachyarrhythmias, as well as daytime conduction and repolarization changes. This narrative review synthesizes current knowledge on ECG manifestations of OSA, encompassing atrial and ventricular ECG characteristics and the burden of supraventricular and ventricular arrhythmias. Emerging evidence suggests that several daytime ECG markers may represent accessible, low-cost indicators of subclinical cardiac remodeling and autonomic imbalance, with potential clinical implications. In addition, there is a rapidly evolving landscape of artificial intelligence applications and wearable-based ECG monitoring for OSA detection and risk stratification. Standardization of ECG-derived markers, validation across diverse populations, and integration into clinical workflows represent key priorities for future research. Recognizing ECG alterations associated with OSA may support earlier diagnosis, improved arrhythmic risk stratification, and more effective multidisciplinary management.

## 1. Introduction and Methods

Obstructive Sleep Apnea (OSA) is characterized by recurrent upper-airway obstruction during sleep, resulting in intermittent hypoxemia, arousals, sympathetic activation, systemic inflammation, and marked negative intrathoracic pressure swings. Although primarily considered a respiratory disorder, OSA is now widely recognized as a major cardiovascular disease modifier, being strongly associated with hypertension, heart failure (HF), stroke, arrhythmias, and increased cardiovascular mortality [1]. The pathophysiological consequences of OSA affect both atrial and ventricular structure and electrophysiology. These alterations are increasingly detectable through standard 12-lead electrocardiographic (ECG) recordings and 24-hour Holter monitoring, which may reveal both daytime conduction or repolarization abnormalities and nocturnal rhythm disturbances [2,3,4,5,6,7]. However, despite its high prevalence, OSA remains widely underdiagnosed. ECG analysis may therefore represent a practical, low-cost tool to improve early recognition of OSA-related cardiac involvement in routine clinical practice.

This work is a narrative review of electrocardiographic abnormalities associated with OSA. The literature was identified primarily through international expert consensus documents and clinical practice guidelines, including statements from the European Heart Rhythm Association/European Society of Cardiology (EHRA/ESC), the American Heart Association/American College of Cardiology (AHA/ACC), the European Respiratory Society (ERS), and the American Academy of Sleep Medicine (AASM). These sources were complemented by focused searches of major cardiovascular and sleep medicine journals, as well as reference lists of key articles.

The reviewed literature spans from foundational electrocardiographic studies to contemporary publications up to 2025, with particular emphasis on original research and expert consensus documents published after 2000.

Studies were included if they: evaluated structural or functional cardiac remodeling in patients with OSA with potential ECG correlates; reported resting or dynamic ECG parameters in OSA populations; investigated mechanistic links between OSA pathophysiology and arrhythmogenesis; assessed the impact of OSA treatment, particularly continuous positive airway pressure (CPAP), on ECG markers or arrhythmic outcomes.

The aim of this review is to provide a comprehensive mechanistic overview of ECG manifestations associated with OSA, while emphasizing their clinical relevance, potential role in arrhythmic risk stratification, and emerging applications in artificial intelligence (AI)-based diagnostic strategies.

## 2. Pathophysiological Mechanisms Linking Obstructive Sleep Apnea Syndrome to Electrocardiographic Alterations

**Figure 1:**
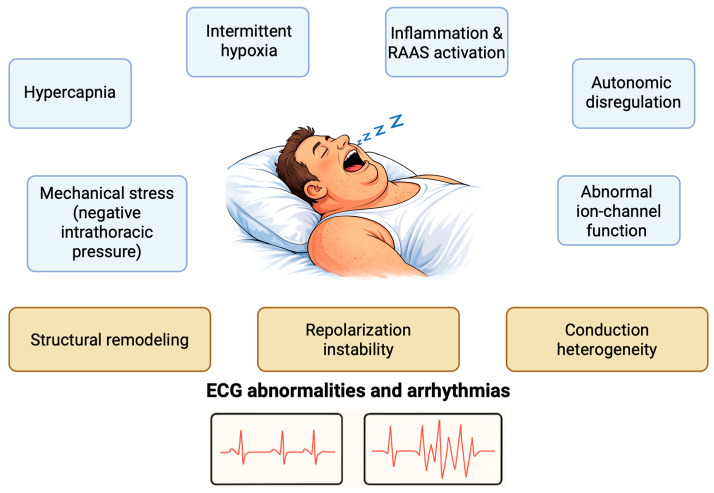
Pathophysiological mechanisms involved in the ECG alterations in patients with OSA. RAAS: Renin-Angiotensin-Aldosterone System.

OSA is a chronic sleep-related disease (SDB), characterized by recurrent pharyngeal obstruction and consequent intermittent disruption of normal ventilation [8]. Several pathophysiological mechanisms act synergistically in OSA to alter cardiac electrophysiology, predisposing patients to both daytime ECG abnormalities and nocturnal arrhythmias (Figure 1). Recurrent respiratory events generate a series of interconnected physiological stressors—including intermittent hypoxia, hypo- and hypercapnia, autonomic dysregulation, gap-junction abnormalities, and marked intrathoracic pressure swings—that converge on three principal myocardial substrates: structural remodeling, conduction heterogeneity, and repolarization instability. These substrates ultimately manifest as a spectrum of atrial and ventricular alterations.

Chronic intermittent hypoxia (CIH), characterized by repetitive cycles of hypoxia and reoxygenation, represents a central upstream driver. CIH promotes oxidative stress through the generation of reactive O_2_ species (ROS) and activation of redox-sensitive transcription factors such as HIF-1α and NF-κB, leading to systemic inflammation, endothelial dysfunction, and myocardial injury [9,10,11]. Over time, these processes result in cardiomyocyte apoptosis and fibrosis, providing the structural substrate for electrical remodeling. On the surface ECG, these changes translate into prolonged P-wave duration, increased P-wave dispersion, PR-interval prolongation, and QTc variability [12,13,14].

Inflammatory activation further amplifies atrial and ventricular remodeling, contributing to supraventricular and ventricular arrhythmias. Elevated circulating cytokines (e.g., IL-6, TNF-α) and activation of the renin–angiotensin–aldosterone system promote fibroblast activation, collagen deposition, and gap-junction remodeling, resulting in conduction heterogeneity and delayed impulse propagation [9,10,15]. Experimental and clinical studies consistently demonstrate that atrial conduction delay and P-wave abnormalities correlate with OSA severity, supporting inflammation as a key mechanistic link between CIH and atrial arrhythmogenesis; in addition, exposure to CIH has been associated with the induction of LV dysfunction [11,13].

In parallel, mechanical and hemodynamic stressors play a pivotal role in atrial remodeling and left atrial enlargement (LAE). Recurrent inspiratory efforts against an occluded upper airway generate large negative intrathoracic pressure swings that acutely stretch the atrial wall. Repetition of this “atrial stretch” promotes structural remodeling and, over time, progressive LAE [16]. Intermittent hypoxemia further exacerbates this process through activation of hypoxia-inducible factor-1 (HIF-1), increased oxidative stress, and cardiomyocyte injury, ultimately contributing to atrial remodeling [17].

Autonomic imbalance represents a key mechanism in OSA, acting both acutely and chronically. Cyclical hypoxemia and hypercapnia stimulate peripheral chemoreceptors and enhance sympathetic outflow, while impaired baroreflex sensitivity reduces autonomic buffering. This imbalance between heightened chemoreflex drive and impaired baroreflex control results in unopposed sympathetic activation, characterized by abrupt surges in heart rate (HR) and transient blood pressure elevations, particularly at the termination of apneic events [18,19,20,21]. Importantly, sympathetic hyperactivation persists during wakefulness in patients with OSA, providing a mechanistic explanation for daytime ECG abnormalities, including reduced HR variability (HRV) and repolarization changes [22,23,24,25]. Conversely, abrupt vagal activation at apnea termination promotes vagal bursts and cyclical sympatho-vagal oscillations, leading to sleep-related bradyarrhythmias and pauses [23,24,25]. At the atrial level, apnea-related activation of intrinsic cardiac ganglionated plexi directly facilitates atrial fibrillation (AF) initiation [26].

Ventricular loading abnormalities induced by obstructive events further impair diastolic relaxation; as the disease progresses, ventricular remodeling, collagen deposition, and myocardial hypertrophy increase left ventricular filling pressures, which are transmitted to the left atrium and contribute to LAE [27,28,29,30].

Beyond hypoxemia, acute hypercapnia directly alters atrial electrophysiology, shortening atrial refractory periods and slowing conduction, thereby further increasing susceptibility to AF [31]. Experimental models also indicate that intermittent hypoxia enhances vulnerability to AF through the combined effects of autonomic imbalance and inflammatory activation [32,33].

At the ventricular level, recurrent airway obstruction, intermittent hypoxemia, arousals, sympathetic surges, and marked intrathoracic pressure swings trigger oxidative stress, abnormal ion-channel function (IKr, IKs, IKur), impaired calcium handling, and mechano-electrical interactions. These processes lead to both electrical and structural remodeling of ventricular myocardium, providing the substrate for depolarization and repolarization abnormalities and predisposing patients to ventricular arrhythmias [34,35].

Repolarization abnormalities observed in OSA—such as QTc prolongation, increased QT dispersion, and prolonged Tpeak-Tend (Tpe) interval—largely reflect the combined effects of autonomic lability and heterogeneous ventricular repolarization [12,36]. Reduced HRV, a hallmark of impaired autonomic regulation, is consistently observed in OSA cohorts and indicates sustained sympathetic predominance with diminished vagal modulation [24,25]. Experimental models of simulated obstructive apneas confirm that even brief episodes of hypoxia and negative intrathoracic pressure increase arrhythmic susceptibility through acute autonomic perturbation and repolarization instability [37].

Mechanical factors further contribute to atrial conduction abnormalities. Atrial stretch induced by large negative intrathoracic pressures during obstructive events, also contributes to conduction abnormalities [38,39]. The resulting mechanical stress triggers electrophysiological remodeling characterized by shortened refractory periods and increased dispersion of conduction. Importantly, expression of connexin-43 (GJA1), a key gap-junction protein, is significantly altered in OSA, further promoting anisotropic conduction and AF susceptibility [40]. The combination of fibrosis, gap-junction remodeling, and altered autonomic tone forms the substrate for both atrial arrhythmias and persistent daytime conduction abnormalities.

Chronic exposure to intermittent hypoxia induces oxidative injury and mitochondrial dysfunction in ventricular myocytes, leading to contractile impairment and electrical remodeling [11]. These changes can manifest on ECG as QRS widening, QT prolongation, and abnormal T-wave morphology, reflecting altered depolarization and repolarization gradients. The presence of nocturnal arrhythmias, including bradycardia, asystole, and ventricular ectopy, is significantly more prevalent in patients with moderate-to-severe SDB [24], likely reflecting acute interactions between hypoxia-induced autonomic surges and an electrically remodeled substrate.

Collectively, the pathophysiological effects of OSA operate on two complementary levels. Acute effects, driven by transient hypoxemia, autonomic surges, and mechanical stress, trigger bradyarrhythmias, pauses, and ectopy during sleep. Chronic effects, mediated by inflammation, fibrosis, and sustained autonomic imbalance, lead to persistent ECG abnormalities and a constant pro-arrhythmic substrate during wakefulness in patients with OSA.

Table 1 provides an overview of the main OSA-related atrial ECG alterations, whereas Table 2 summarizes the OSA-related ventricular ECG alterations.

## 3. Atrial Electrocardiographic Alterations in Obstructive Sleep Apnea Syndrome

As discussed above, both the acute and chronic consequences of obstructive apneas exert profound effects on atrial structure and electrophysiology, giving rise to distinct ECG manifestations. Acutely, transient hypoxemia caused by upper-airway collapse has been hypothesized to alter the atrial effective refractory period, thereby promoting electrical instability [44]. Chronically, the hemodynamic consequences of OSA include increased left atrial pressure and subsequent LAE [45]. Over time, sustained atrial pressure overload promotes a pro-inflammatory and pro-thrombotic milieu, ultimately resulting in fibrotic remodeling of the atrial myocardium [46].

### 3.1. P-Wave Modifications

In physiological conditions, atrial electrical activation begins with the generation of an impulse in the atrial pacemaker located in the sinoatrial node area. Once initiated, the impulse rapidly propagates along the crista terminalis and proceeds anteriorly toward the lower portion of the right atrium. In most individuals, left atrial activation is initiated predominantly through conduction across Bachmann’s bundle, which extends from the anterior right atrium-superior to the fossa ovalis-to the left atrium near the right superior pulmonary vein. Simultaneously, activation spreads across the interatrial septum, encircling the fossa ovalis to reach the superior portion of the interventricular septum. The last region to be depolarized is typically located over the inferolateral surface of the left atrium.

Thus, right atrial activation begins earlier than that of the left atrium, while left atrial activation continues after completion of right atrial activation. This pattern of atrial activation—initiated in the superior right atrium and spreading simultaneously leftward toward the left atrium and inferiorly toward the atrioventricular (AV) node—underlies the normal P wave morphology observed on the standard 12-lead ECG [47].

To better understand the alterations observed in OSA, it is important to recall that the parameters defining a “physiological” P wave can be summarized as follows:

Duration: a normal P wave has a duration of less than 120 ms. A prolongation beyond this threshold suggests delayed conduction along Bachmann’s bundle, consistent with partial interatrial block [48]. In advanced interatrial block, the left atrium is not activated via Bachmann’s bundle; instead, depolarization occurs retrogradely through the myocardium surrounding the AV node. This results in a P-wave duration >120 ms with a biphasic morphology in the inferior leads (II, III, aVF) [49].

Dispersion: defined as the difference between the maximum and minimum P-wave duration across the 12 leads. Values >40 ms are considered abnormal. Increased dispersion has been shown to predict occurrence and recurrence of supraventricular tachyarrhythmias, particularly AF [50,51,52].

Amplitude: normally exceeds 0.1 mV. Reduced P-wave amplitude in lead I has been associated with interatrial conduction abnormalities and recurrence of paroxysmal AF following catheter ablation [41].

Area: calculated as the semiproduct of duration and amplitude. Values greater than 4 ms·mV are typically associated with LAE [53].

Axis: the normal P-wave axis averages approximately +60°, usually ranging between 0° and +75° [47].

P-wave terminal force in V1 (PTFV1): a small terminal negative deflection of the P wave in lead V1 may be present in normal ECGs; however, a depth > 0.1 mV in depth and duration > 0.04 s is indicative of LAE, as originally described by Morris et al. [54].

Moderate-to-severe OSA is recognized as an independent factor contributing to atrial remodeling and LAE [55]. The anatomical and structural alterations affecting the cardiac chambers inevitably manifest on the standard 12-lead ECG.

The pathophysiology underlying OSA-related LAE is multifactorial and includes the mechanisms described above.

These changes ultimately increase left ventricular filling pressures, which are transmitted to the left atrium, contributing to its progressive dilation.

Based on current evidence, specific alterations in atrial depolarization can be identified in the 12-lead ECG of patients with OSA:

Prolonged P-wave duration (>120 ms): considered a reliable marker of LAE [56].

Increased PTFV1: characterized by a wide and deep terminal negative component (>0.04 s × >1 mm), indicative of left atrial overload.

P-wave dispersion >40 ms: reflects heterogeneous atrial conduction and predicts an increased risk of AF recurrence [56,66].

Increased P-wave area: a study by Arias et al. [27] demonstrated that, in patients with paroxysmal AF and OSA, the signal-averaged positive P-wave area is significantly greater during respiratory events compared with wakefulness. This finding likely reflects the acute effects of increased venous return and right atrial volume during apneic episodes.

### 3.2. PR Interval Modifications and Atrioventricular Block

The PR segment represents the isoelectric interval between the end of the P wave and the onset of the QRS complex and forms part of the PR interval, which extends from the beginning of P wave to the onset of the QRS complex. In adults, a normal PR interval ranges between 120 and 200 ms, and is commonly measured in the lead with the shortest duration to avoid misinterpretation in the presence of pre-excitation.

Electrophysiologically, the PR interval reflects atrial depolarization, conduction delay within the AV node, and propagation through the His–Purkinje system until sufficient ventricular myocardium is activated to generate the QRS complex. Its isoelectric appearance on the surface ECG is explained by the low-amplitude potentials of the conduction system, which can only be detected using intracardiac recordings [47].

In the context of OSA, alterations of the PR interval have been reported; however, the clinical interpretation of PR prolongation requires careful consideration of potential confounders (e.g., age, medications, and underlying conduction disease). In a study by Karamanlı et al. [67], 85 patients were stratified according to OSA severity based on ECG and polysomnography (PSG) findings. A significant prolongation of the PR interval was observed in those with severe OSA compared with patients with mild to moderate disease. The mechanisms underlying delayed AV conduction and AV block may include LAE and structural remodeling as well as autonomic imbalance characterized by sympathetic predominance during sleep and transient surges in vagal activity during arousal episodes. 

In a study involving 16 patients with OSA and no baseline electrophysiological abnormalities of AV conduction or sinus node function, Koehler et al. [68] reported a total of 651 episodes of AV block at PSG, most of which occurred during REM sleep and were significantly associated with apneic/hypopneic events accompanied by ≥4% O_2_ desaturation. Notably, treatment with CPAP or Bilevel Positive Airway Pressure (BiPAP) led to a marked reduction in both the apnea–hypopnea index (AHI) and the frequency of bradyarrhythmic events. However, cases have been reported in the literature in which advanced AV block appears resistant to treatment. Kawana et al. described a 67-year-old man with severe OSA who developed a 2:1 AV block exclusively during the phasic events of REM sleep; notably, CPAP therapy did not resolve the conduction abnormality [69]. Conversely, cases have been reported in which CPAP therapy improved nocturnal ECG abnormalities in the presence of advanced AV block. Maeno et al. described a 54-year-old man with severe OSA who exhibited advanced AV block with ventricular asystole lasting more than 6 s during PSG, notably occurring prior to O_2_ desaturation. A previously performed electrophysiological study had demonstrated no abnormalities in AV conduction or His–Purkinje system function. In this case, resolution of OSA with CPAP therapy led to marked improvement of the advanced AV block, supporting the interpretation that the bradyarrhythmia was an OSA-induced conduction disturbance occurring independently of desaturation [70].

## 4. Supraventricular Tachyarrythmias

OSA has been associated with both bradyarrhythmic and tachyarrhythmic events. The arrhythmogenic substrate of OSA arises not only from atrial and ventricular remodeling with subsequent fibrosis, but also—more prominently—from intermittent hypoxia, enhanced sympathetic activation, and mechanical stress [63,71,72,73,74]. For clarity, the relationship between OSA and supraventricular arrhythmogenesis will be discussed under two subheadings: supraventricular ectopy and AF.

### 4.1. Supraventricular Ectopy

In a 2021 study, Kawano et al. investigated the relationship between OSA and supraventricular ectopy in 431 patients who underwent both PSG and 24 h Holter ECG monitoring. Participants were stratified into four groups according to their AHI quartiles. The study demonstrated a significant difference in the frequency of premature supraventricular contractions (PSVCs) per hour of sleep among the groups. Multivariate logistic regression analysis further revealed that patients in the highest AHI quartile were significantly more likely to exhibit ≥5 PSVCs per hour during sleep, whereas no significant differences were observed in PSVCs frequency during wakefulness [42].

In another study, Quintal et al. [75] evaluated 233 patients stratified by OSA severity (89 mild, 74 moderate, and 70 severe). In that study, OSA severity was associated with an increased prevalence of frequent premature ventricular contractions (PVCs), whereas no significant association was found with PSVCs.

Horváth et al. [43] explored the association between OSA and supraventricular ectopy in a cohort of 656 patients with HF with reduced ejection fraction (HFrEF), including 430 with OSA, 150 with central sleep apnea (CSA), and 76 control subjects [43]. Combined PSG and ECG analyses demonstrated that the prevalence of excessive supraventricular ectopic activity—defined as ≥30 PSVCs per hour or supraventricular tachycardia (SVT) lasting ≥20 beats—was markedly higher in patients with OSA and CSA compared with controls (0%, 9%, and 12%, respectively). Interestingly, in the same population, statistical analyses revealed no significant differences in AF burden or in ventricular ectopy across the groups.

The differences between types of arrhythmias in patients with or without HFrEF suggest that impaired ventricular function may contribute to the apparent discrepancy between these findings. Such divergence underscores the need for further studies to clarify the complex interplay among OSA, supraventricular ectopy, and ventricular dysfunction.

### 4.2. Atrial Fibrillation

In September 2024, the ESC released updated guidelines on the management of AF, introducing the CARE-AF framework, which emphasizes: *C: comorbidity management, A: avoiding stroke, R: rate and rhythm control, and E: (re-)evaluation*. This integrated approach underscores the importance of addressing the underlying pathophysiological substrates of AF, within which OSA plays a central and well-recognized role [76].

OSA is highly prevalent among patients with AF [77,78]; however, optimal screening strategies for OSA in this population remain uncertain. PSG or home sleep apnea testing are currently preferred over symptom-based questionnaires, which often lack sensitivity and may underestimate disease burden [77,79,80,81].

Untreated OSA has been consistently associated with poorer AF outcomes following cardioversion or catheter ablation, whereas treatment with CPAP has been shown to reduce AF recurrence rates and improve rhythm control [57,65,82,83,84,85].

Nevertheless, data from RCTs remain heterogeneous, and evidence for a definitive cardiovascular mortality benefit from OSA treatment in AF patients is still inconclusive [58,59,60,86].

## 5. Ventricular Electrocardiographic Alterations in Obstructive Sleep Apnea

### 5.1. Normal Ventricular Conduction and Repolarization

In healthy adults without cardiac disease, the QRS complex duration (ventricular depolarization) typically ranges from 80 ms to approximately 110–120 ms. It has been observed that, on average, women tend to exhibit slightly shorter QRS durations compared to men. However, this discrepancy in QRS duration cannot be fully attributed to differences in heart size alone [61].

Clinically, a QRS duration of ≥120 ms is generally considered prolonged (indicative of bundle branch block or intraventricular conduction delay), whereas very short QRS durations (<70 ms) are rare and generally considered without clinical significance in a healthy population.

The frontal QRS axis (i.e., the mean electrical axis in the limb leads) is typically oriented between approximately −30° and +90° in an adult. An axis within this range is considered “normal” (pointing downward and leftward, reflecting normal left ventricular dominance). Deviations beyond these limits are indicative of left axis deviation (e.g., −45°) or right axis deviation (e.g., +110°), which may suggest underlying pathology if pronounced. In summary, a QRS axis that extends from the 11 o’clock to the 3 o’clock position on the vector circle (−30° to +90°) is within the normal physiological range in adults [62].

### 5.2. QT Interval and Corrected QT (QTc)

The QT interval measures ventricular depolarization and repolarization time. As QT varies with HR, the focus in clinical practice is on the HR-corrected QT (QTc), for example, using Bazett’s formula. In healthy adults, normal QTc values are around 400 ± 20 ms, with upper limits differing by sex. Following puberty, a QTc > 450 ms in men or >460 ms in women is frequently deemed borderline prolonged.

According to epidemiological data, the 99th percentile of the QTc interval is ~470 ms for men and ~480 ms for women. Sustained QTc values at or beyond 480 ms are indicative of a cardiac abnormality, irrespective of gender. Clinically, a QTc ≥500 ms is widely recognized as markedly prolonged and associated with high arrhythmic risk, especially torsades de pointes ventricular tachycardia [64].

### 5.3. Tpeak-Tend Interval (Tpe) and Tpe/QT Ratio

The Tpe interval, measured from the peak to the end of the T wave, is an index of transmural dispersion of repolarization. In healthy adults, Tpe values are typically 60–70 ms. The upper limit of normal Tpe is ~90 ms (95% confidence upper bound), and ~100 ms when HR-corrected (Tpe-c). Prolonged Tpe has been linked to greater repolarization dispersion and increased arrhythmic risk (e.g., reentrant ventricular arrhythmias), although there is not yet an official consensus cutoff for risk stratification [87].

Tpe usually shortens or lengthens in parallel with QT. Thus, the Tpe/QT ratio (or Tpe/QTc), is assessed as a HR-independent marker of repolarization heterogeneity. In healthy individuals, this ratio averages around 0.18. The hypothesis that elevated peak-to-trough ratios serve as markers of arrhythmia risk has been proposed [88].

### 5.4. Ventricular Depolarization (QRS Complex) Abnormalities

Several clinical studies have documented distinctive QRS complex alterations in patients with OSA. In a prospective cohort (n = 193 patients), increasing OSA severity was associated with a progressive leftward QRS axis shift, low QRS voltages, and a higher prevalence of fragmented QRS (fQRS). These findings reflect a sequence of progressive depolarization abnormalities and electrical remodeling [89].

Cross-sectional studies also demonstrate a graded relationship between OSA severity and QRS duration. In one study (n = 221 patients), mean QRS duration increased across AHI categories, with QRS ≥ 100 ms occurring almost exclusively in severe OSA. This association was particularly evident in women [90].

Earlier clinical observations described deep S waves in lateral precordial leads (V5-V6) and RS patterns with deep S in limb leads among OSA patients, suggesting delayed depolarization of the right ventricular outflow tract and/or left anterior fascicular block [5].

A contemporary sleep clinic cohort confirmed these findings, identifying deep S in V5-V6 and RS patterns with deep S in I/AVF as ECG features of OSA on univariate analysis [6].

Mechanistically, OSA-related LV hypertrophy, fibrosis, right-sided strain, and conduction heterogeneity underlie these QRS alterations—including QRS prolongation, low voltage, axis deviation, and fragmentation—which collectively mark increased arrhythmic vulnerability and a higher risk of sudden cardiac death [35].

It is noteworthy that depolarization abnormalities in OSA often occur in the presence of only modest QRS prolongation (e.g., 100–120 ms). However, findings such as low voltage, axis shift, and fragmented QRS indicate more advanced structural remodeling than QRS duration alone would suggest [71,91].

### 5.5. Ventricular Repolarization (QT/QTc, Tpe) Abnormalities

ECG studies consistently show that OSA is associated with abnormalities of ventricular repolarization. In a clinical study comparing patients with sleep apnea-hypopnea syndrome (SAHS) (including obstructive apneas, central apneas and hypopneas) and healthy controls, SAHS patients exhibited prolonged QTc intervals, increased Tpeak-Tend (Tpe) duration, and elevated Tpe/QT and Tpe/QTc ratios across various sleep stages. These indices reflect enhanced transmural dispersion of repolarization and heightened susceptibility to ventricular arrhythmias [7].

Arousals in OSA are characterized by abrupt autonomic fluctuations accompanied by RR and QT interval shortening and PR prolongation. Moreover, QT changes correlate with the degree of preceding O_2_ desaturation, indicating dynamic and labile repolarization at the termination of respiratory events [92].

Mechanistic reviews emphasize that intermittent hypoxia increases ROS and disrupts ion-channel function, particularly through reduced repolarizing K^+^ currents, leading to prolonged action potential duration and amplified repolarization heterogeneity [34,35].

When interpreting OSA-related repolarization abnormalities in the context of QTc and Tpe thresholds derived from general-population data, it is noteworthy that even modest increases in QTc (e.g., into the 450–480 ms range) or Tpe/QT may hold clinical significance. This is because they reflect an augmentation of repolarization dispersion superimposed on nocturnal hypoxemia and autonomic surges [61,93].

Among QTc and Tpe-based indices in OSA, QTc is currently the most clinically actionable parameter, while Tpe remains mainly research or “extended” risk-phenotyping tool without guideline-level validation for routine screening or risk stratification.

### 5.6. Ventricular Arrhythmias

Observational studies consistently document a higher prevalence of PVCs and non-sustained ventricular tachycardia (NSVT) in patients with OSA. In a retrospective analysis of sleep-laboratory data from 273 patients with OSA, ECG abnormalities were identified in 10.6% of cases. The most common abnormality was PVCs, representing approximately 76% of all detected events. Patients with ECG abnormalities tended to be older and more likely to have hypertension [4].

Data from broader OSA cohorts indicate that cardiac arrhythmias of any type may occur in 30–60% of patients, with PVCs reported in up to two-thirds of individuals—particularly during sleep and in close temporal association with apneic events [71,91].

In the Akershus Sleep Apnea Project, middle-aged subjects with predominantly mild–moderate, often unrecognized OSA showed a higher prevalence of frequent PVCs (>5/hour) during both nighttime (12.2% vs. 4.7%) and daytime (14% vs. 5.1%) periods compared with controls. The AHI remained independently correlated with ventricular ectopy after adjustment for confounders [94].

Large cohort studies demonstrate a consistent pattern of nocturnal ventricular arrhythmias and sudden cardiac death occurring during sleep in patients with OSA. This provides compelling evidence for a causal link between discrete respiratory events and the triggering of a vulnerable arrhythmic substrate [34,92,93,95].

More recently, implantable loop recorder (ILR) studies in OSA patients without known arrhythmias have revealed a substantial prevalence of previously unrecognized atrial and ventricular tachyarrhythmias, as well as clinically relevant bradyarrhythmias and pauses. These findings underscore that conventional short-term monitoring strategies may significantly underestimate the true arrhythmic burden in this population [95].

Notably, arrhythmic risk increases in parallel with the severity and depth/duration of nocturnal desaturation. This may help explain why ventricular ectopy and NSVT are particularly prevalent in severe OSA and in patients with coexisting structural heart disease [96].

Temporal analyses of clinical datasets demonstrate a close association between apneic events and arrhythmic episodes, supporting the hypothesis that OSA may elevate the risk of sudden cardiac death during sleep. The likely mechanism involves acute autonomic surges and mechanical stress occurring at apnea termination, which together may trigger ventricular arrhythmias [34,92].

### 5.7. Therapeutic and Translational Notes

Randomized clinical trials have shown that CPAP therapy reduces sympathetic activity and oxidative stress—key mechanisms underlying electrical instability—thus providing biological plausibility for a reduction in arrhythmic risk. Clinical evidence suggests that OSA treatment may decrease the frequency of ventricular arrhythmias; however, definitive randomized outcome data demonstrating a benefit on hard cardiovascular outcomes remain limited [34].

Evidence suggests that CPAP consistently reduces nocturnal supraventricular and ventricular ectopy and improves indices of autonomic balance. However, data from randomized trials on AF burden are mixed and do not support a stand-alone antiarrhythmic effect, underscoring the heterogeneity of OSA phenotypes and the critical importance of adequate treatment adherence [71,91,93,95,96]. However, in this context, observational studies have suggested that individuals with OSA not treated with CPAP respond poorly to treatments for AF, with a higher risk of recurrence after cardioversion or catheter ablation [82]. Conversely, CPAP-treated patients appear to partially mitigate their propensity toward AF development and recurrence [76].

CPAP therapy in patients with OSA has been consistently associated with a reduction in sleep-related bradyarrhythmias, including sinus pauses and high-grade atrioventricular block, in many cases obviating the need for permanent pacing when OSA is adequately treated. Data derived from ILR monitoring further suggest that, rather than completely eliminating atrial or ventricular tachyarrhythmias, CPAP may be particularly effective in suppressing sleep-related bradyarrhythmias and prolonged pauses. This observation supports the concept that OSA treatment can meaningfully modify—but not fully abolish—arrhythmic risk [95].

Importantly, because the reduction in nocturnal arrhythmic burden achieved with CPAP therapy is adherence-dependent, suboptimal CPAP adherence represents a major limitation in the management of OSA-related arrhythmias and may account for the persistence of arrhythmic burden despite treatment initiation [97].

Overall, CPAP should be considered a key component of rhythm management in OSA, particularly for bradyarrhythmias clearly linked to apneic events, while its impact on tachyarrhythmias (especially recurrent AF) appears more modest and is best interpreted within a comprehensive cardiovascular risk-factor modification strategy.

## 6. Artificial Intelligence

As reported above, the link between OSA and elevated cardiovascular risk is well established; therefore, early diagnosis is crucial to enable timely and personalized treatment. Nevertheless, an estimated 85–95% of OSA cases remain undiagnosed. The gold standard for diagnosis—overnight PSG—provides comprehensive monitoring of physiological parameters during sleep but is resource-intensive, time-consuming, costly, and often limited in accessibility and practicality for routine clinical use [98].

In this context, AI-based detection techniques have been proposed in the scientific literature to address the limitations of PSG and to facilitate automated OSA screening and detection. AI algorithms are capable of rapidly and accurately identifying apnea-related patterns, playing a crucial role both in clinical settings—such as continuous monitoring of at-risk patients or real-time surveillance during surgical procedures—and enabling home-based screening through wearable technologies [99].

Emerging computational approaches using ECG signals—and, in some cases, pulse oximetry—have demonstrated the capacity to identify apnea-related physiological patterns. Machine learning (ML) algorithms leveraging RR-interval dynamics and O_2_ saturation (SpO_2_) fluctuations have achieved high diagnostic accuracy, highlighting the potential role of ECG-derived ventricular and autonomic markers in the detection and risk stratification of OSA [100].

AI and ML models for OSA detection demonstrate high diagnostic accuracy; however, they cannot currently replace conventional diagnostic methods and should be regarded as assistive tools, as PSG remains the diagnostic gold standard. A major challenge lies in the need for rigorous external validation across heterogeneous populations to ensure robustness, generalizability, and clinical reliability. Regulatory approval pathways, the risk of false-positive results and overdiagnosis, and integration into real-world clinical workflows represent additional barriers to widespread implementation.

Nevertheless, as AI and ML technologies continue to evolve, these systems are expected to become increasingly accurate and autonomous, potentially enabling large-scale screening and real-time monitoring of OSA using simple, non-invasive tools such as the ECG. If appropriately validated and integrated, this development could substantially enhance early detection and management of a highly prevalent yet frequently underdiagnosed condition.

AI is reshaping the diagnostic approach to OSA, with a particular focus on ML and deep learning (DL) techniques. ML algorithms learn from structured clinical data using handcrafted features, whereas DL models can automatically extract complex patterns directly from raw physiological signals such as ECG recordings. Both approaches have demonstrated high diagnostic accuracy and hold substantial potential to complement conventional diagnostic pathways [101].

### 6.1. Wearable Devices

A promising solution to overcome the diagnostic challenges of OSA is wearable AI, which leverages AI algorithms and techniques to collect and analyze data, such as HR, respiration rate, and SpO_2_. The devices utilized—including smartwatches, fitness trackers, and smart glasses—are highly accessible, reliable, and objective, and enable continuous, real-time monitoring, offering a practical alternative to traditional diagnostic methods; the detection of physiological parameters through the use of wearable devices not only enables the identification of potential respiratory disorders such as OSA but also facilitates disease classification and severity estimation [102].

Considering the significant association between OSA severity and ECG abnormalities, several wearable ECG-based monitoring systems have been developed. Baty et al. [103] introduced a novel wearable ECG belt specifically designed for sleep monitoring, capable of acquiring single-lead ECG signals during overnight recordings. By extracting HRV and ECG-derived features, the device enabled accurate classification of OSA severity in a home-based setting, demonstrating that dedicated wearable ECG sensors may represent a feasible and cost-effective alternative to conventional sleep studies for screening and risk stratification.

Similarly, Bsoul et al. [104] proposed *ApneaMedAssist*, a fully automated system based on single-lead nocturnal ECG acquisition, combined with machine learning classification and smartphone-based processing to detect apnea episodes in real time. The lightweight ECG sensor allows continuous and prolonged ECG monitoring, supporting both home-based screening and perioperative evaluation, and may assist in treatment optimization, including CPAP adjustment.

#### Computer-Aided Diagnosis

When OSA is suspected, ECG monitoring—either using the conventional 12-lead ECG or, as discussed in the previous section, through emerging AI-assisted solutions—is an essential component of the diagnostic workup.

The standard 12-lead ECG is a readily available, non-invasive, and cost-effective tool that can aid in the early detection of subclinical cardiac involvement in patients with OSA. This has important clinical implications, as it allows the identification of individuals at increased arrhythmic risk and facilitates cardiovascular risk stratification, thereby guiding timely therapeutic interventions. Moreover, the presence of characteristic ECG abnormalities may serve as a useful surrogate marker for suspecting OSA, a condition that remains widely underdiagnosed despite its substantial impact on cardiovascular health. These considerations support the integration of ECG analysis into the routine evaluation of patients at risk for SDB, with the aim of improving early detection and management strategies.

Nevertheless, detecting OSA events based solely on ECG data remains challenging. Reliable diagnosis requires prolonged observation, and manual interpretation is prone to errors and subject to both inter- and intra-observer variability. In this context, AI-driven computer-based algorithms may assist in ECG signals interpretation, enabling diagnosis with a high degree of accuracy [105].

Computer-Aided Diagnosis (Co-AD) systems apply data mining and ML techniques to determine whether a given ECG signal sequence exhibits features suggestive of OSA. Sheta et al. [98] proposed a Co-AD system consisting of three main stages for the automated detection of apnea events based on ECG variations: signal preprocessing to remove noise (e.g., using a notch filter), feature extraction to identify critical ECG characteristics associated with apnea, and ML-based classification to determine the presence or absence of OSA.

The integration of Co-AD systems into clinical practice holds significant promise, as it may enhance diagnostic efficiency, reduce human error, and facilitate the early identification of at-risk patients, even in resource-limited settings.

### 6.2. Future Perspective

AI-based detection techniques have been proposed in the scientific literature to address the limitations associated with PSG and to facilitate the automatic detection of OSA. AI algorithms are capable of rapidly and accurately identifying apneic events, playing a crucial role both in clinical settings—such as continuous monitoring of at-risk patients or real-time surveillance during surgical procedures—and enabling home-based screening through the deployment of previously discussed wearable technologies [99]. AI models for detecting OSA demonstrate high diagnostic accuracy; nevertheless, to date, they cannot replace the traditional diagnostic methods and can only assist in the identification of potential sleep disorders, as PSG remains the gold standard. Future research should focus on validating AI-based models for OSA detection across heterogeneous populations to improve their robustness, generalizability, and clinical applicability. Expanding their use as diagnostic support tools could enable the early identification of individuals with a high pre-test probability of OSA, allowing for timely intervention and potentially reducing the risk of associated complications.

As AI and ML techniques continue to evolve, these systems are expected to become increasingly accurate and autonomous, potentially allowing for large-scale screening and real-time monitoring of OSA using simple, non-invasive tools such as the ECG. This could represent a major advancement in the early detection and management of a condition that is both highly prevalent and frequently underdiagnosed.

## 7. Clinical Implications

Recognizing ECG manifestations of OSA has important clinical implications beyond arrhythmia detection. Routine ECG parameters—traditionally considered nonspecific—may serve as accessible, low-cost markers of disease severity and cardiovascular risk.

Atrial abnormalities such as prolonged P-wave duration, increased P-wave dispersion, and abnormal PTFV1, reflect early atrial remodeling and may identify patients at risk for AF even in the absence of symptoms. Similarly, PR-interval prolongation and intermittent AV block documented on ambulatory ECG recordings may signal autonomic imbalance or structural atrial disease related to longstanding OSA.

Ventricular alterations are characterized by ventricular depolarization abnormalities, QRS widening, axis deviation, low voltage, fragmented QRS (fQRS), repolarization abnormalities, and increased QTc- and Tpe-based indices, together with arousal-related changes. These severity-dependent alterations reflect intermittent hypoxia, autonomic activation, and mechanical loading [34]. These alterations serve as noninvasive markers of ventricular remodeling, heightened repolarization heterogeneity, and increased arrhythmic susceptibility, and may help stratify patients who could benefit from risk-factor optimization, closer rhythm monitoring, or CPAP therapy.

Daytime ECG features such as prolonged or fragmented QRS and axis shift may indicate subclinical ventricular remodeling, whereas nocturnal ECG changes surrounding apneic episodes capture dynamic arrhythmogenic triggers.

CPAP therapy, when indicated, targets upstream drivers of electrical instability—specifically sympathetic overactivity and oxidative stress—and may therefore mitigate arrhythmic risk, although definitive outcome data remain limited [60]. ECG findings may also assist in evaluating treatment response. Improvement in autonomic tone and repolarization stability following CPAP has been documented, suggesting a role for ECG-based monitoring to assess adherence and physiological benefits.

Finally, integrating ECG data with AI-driven algorithms and wearable-based monitoring systems may significantly enhance early detection of SDB, identifying high-risk individuals thereby reducing underdiagnosis and enabling earlier intervention.

Overall, ECG analysis provides a practical, scalable, and widely available method for improving cardiovascular risk stratification and guiding personalized management strategies in patients with OSA.

## 8. Conclusions

OSA induces a spectrum of atrial and ventricular ECG abnormalities that reflect both acute physiological stress and chronic structural remodeling. These abnormalities provide accessible, non-invasive markers of autonomic imbalance and arrhythmic susceptibility.

Recognizing these ECG signatures can improve early identification of high-risk patients and may guide clinical decisions in AF management, ventricular arrhythmias, and bradyarrhythmias. However, standardized ECG-based markers, prospective validation, and integration into clinical workflows remain unmet needs.

AI and wearable ECG technologies offer promising tools for detection and risk stratification but require rigorous validation. Incorporating ECG analysis into routine cardiovascular assessment has the potential to enhance early diagnosis, personalize care, and reduce the arrhythmic burden associated with OSA.

## Figures and Tables

**Table 1:** Summary of atrial ECG alterations associated with OSA, and their underlying mechanisms. The alterations include atrial conduction alterations and arrhythmias. These changes result primarily from intermittent hypoxia, autonomic dysregulation, and cardiac remodeling.

ECG Parameter	Normal Range	Abnormal Findings in OSA	Clinical Significance
P-wave duration [6,7,8]	<120 ms	>120 ms; biphasic morphology in inferior leads (advanced interatrial block)	Marker of delayed interatrial conduction; associated with atrial remodeling and AF risk
P-wave dispersion [9,10,11]	<40 ms	>40 ms	Predictor of supraventricular arrhythmias, particularly AF recurrence
P-wave amplitude [12]	>0.1 mV	Reduced amplitude in lead I	Linked to interatrial conduction abnormalities and recurrence of paroxysmal AF post-ablation
P-wave area [13]	≤4 ms·mV	>4 ms·mV	Associated with LAE
P-wave axis [5]	~60° (0–75°)	Outside physiological range	Suggests atrial conduction abnormalities
P-wave terminal force in V1 (PTFV1) [14]	Small terminal negative deflection	Depth >0.1 mV and duration >0.04 s	Indicative of LAE; linked to OSA-induced atrial remodeling
PR interval [29]	120–200 ms	Prolonged PR interval; first-degree atrioventricular block	Reflects atrial remodeling, autonomic imbalance, and vagal surges during apneic events
Atrioventricular block (AVB) [41]	Normal AV conduction	Transient or persistent AVB (often during REM sleep); may improve with CPAP	Demonstrates OSA-induced conduction disturbance; variable CPAP response
Supraventricular ectopy [36,37,38]	Occasional PSVCs	Increased PSVCs ≥5/h [42]; excessive ectopy ≥30 PSVCs/h or SVT ≥20 beats [43]	Highlights OSA-related supraventricular arrhythmogenicity; interplay with ventricular dysfunction
Atrial fibrillation [39,40,41,44,45,46,47,48,49,50,51,52,53,54,55,56]	Sinus rhythm	Increased prevalence and recurrence in untreated OSA; inconsistent benefit of CPAP in RCTs	OSA recognized as major modifiable substrate in AF pathophysiology

AF: atrial fibrillation, AVB: atrio-ventricular block, CPAP: continuous positive airway pressure, ECG: electrocardiogram, LAE: left atrial enlargement, PSVCs: premature supra-ventricular contractions, RCTs: randomized clinical trials, REM: rapid eye movement.

**Table 2:** Summary of ventricular ECG alterations associated with OSA, and their underlying mechanisms. The alterations include ventricular conduction alterations and arrhythmias.

ECG Parameter	Normal Range	Abnormal Findings in OSA	Clinical Significance
QRS axis [57,58,59,60]	Frontal QRS axis typically around −30° to +90°	Progressive leftward shift of the frontal QRS axis with increasing OSA severity	Reflects electrical remodeling and altered ventricular depolarization sequence
QRS voltage [57,58,59,60]	Normal amplitude in limb leads ≥5 mm, precordial ≥10 mm	Low QRS voltages in limb and/or precordial leads	Suggests myocardial fibrosis, edema, or conduction heterogeneity due to OSA-induced remodeling
Fragmented QRS (fQRS) [58]	Absent in normal ECG	Increased prevalence in OSA (more frequent with severity)	Marker of myocardial scarring and arrhythmic vulnerability
QRS duration [59]	<100 ms (normal range)	Prolonged QRS (≥100 ms), especially in severe OSA and female patients	Indicates conduction delay, possibly from LV hypertrophy or fibrosis
S waves in V5–V6/limb leads [60]	Normally small or absent S wave in V5–V6	Deep S waves in V5–V6 and RS patterns in I/AVF	Suggests late depolarization of RV outflow tract or left anterior fascicular block
QTc interval [61]	Men: <450 ms; Women: <460 ms	Prolonged QTc in OSA patients	Reflects delayed ventricular repolarization; increases risk of malignant arrhythmias
Tpe interval (T peak–T end) [61]	Typically < 100 ms	Prolonged Tpe and increased Tpe/QT, Tpe/QTc ratios	Indicates increased transmural dispersion of repolarization and arrhythmia susceptibility
Dynamic QT changes during arousal [62]	Stable QT interval	QT shortening and PR lengthening during arousal; correlated with O_2_ desaturation	Reflects autonomic instability and dynamic repolarization lability during apneic events
Premature ventricular contractions (PVCs) [63]	Rare (<1% of beats)	Increased PVCs (most common abnormality, ~76% of ECG changes in OSA)	Marker of ventricular irritability and possible precursor to VT/VF
Non-sustained ventricular tachycardia (NSVT) [64]	Absent	Higher prevalence in OSA, especially nocturnal	Associated with increased risk of sudden cardiac death, particularly during sleep
Temporal relation of arrhythmias [65]	Absent	Arrhythmias temporally linked to apneic events	Suggests autonomic surges and mechanical stress as triggers of ventricular arrhythmias

HRV: heart rate variability, NSVT: non-sustained ventricular tachycardia, O_2_: oxygen, PVCs: premature ventricular contractions, QTc: corrected QT interval, RV: right ventricular, Tpe: Tpeak-to-Tend interval, VT/VF: ventricular tachycardia/ventricular fibrillation.

## Data Availability

No new data were created or analyzed in this study.

## References

[1] Yeghiazarians Y., Jneid H., Tietjens J.R., Redline S., Brown D.L., El-Sherif N., Mehra R., Bozkurt B., Ndumele C.E., Somers V.K. (2021). Obstructive Sleep Apnea and Cardiovascular Disease: A Scientific Statement From the American Heart Association. Circulation.

[2] Otto M.E., Belohlavek M., Romero-Corral A., Gami A.S., Gilman G., Svatikova A., Amin R.S., Lopez-Jimenez F., Khandheria B.K., Somers V.K. (2007). Comparison of cardiac structural and functional changes in obese otherwise healthy adults with versus without obstructive sleep apnea. Am. J. Cardiol..

[3] Romero-Corral A., Somers V.K., Pellikka P.A., Olson E.J., Bailey K.R., Korinek J., Orban M., Sierra-Johnson J., Kato M., Amin R.S. (2007). Decreased right and left ventricular myocardial performance in obstructive sleep apnea. Chest.

[4] Bhatia M., Khatiwada S., Dubey G. (2017). Electrocardiographic Abnormalities in Obstructive Sleep Apnea: A Retrospective Study. Sleep Vigil..

[5] Khalil M.M., Rifaie O.A. (1998). Electrocardiographic changes in obstructive sleep apnoea syndrome. Respir. Med..

[6] Shankar S., Gupta S.S., Rojas-Marte G., Demir S., Saxena A., Obiagwu C., Aggarwal N., Rai A.K., Kamholz S., Shetty V. (2019). Electrocardiographic Associations Seen with Obstructive Sleep Apnea. Sleep Disord..

[7] Zeng L., Liang J., Liao Y., Zhou G., Zhang X., Luo Y. (2019). Variation of Electrocardiogram Features Across Sleep Stages in Healthy Controls and in Patients with Sleep Apnea Hypopnea Syndrome. Int. Heart J..

[8] Tzeis S., Gerstenfeld E.P., Kalman J., Saad E.B., Sepehri Shamloo A., Andrade J.G., Barbhaiya C.R., Baykaner T., Boveda S., Calkins H. (2024). 2024 European Heart Rhythm Association/Heart Rhythm Society/Asia Pacific Heart Rhythm Society/Latin American Heart Rhythm Society expert consensus statement on catheter and surgical ablation of atrial fibrillation. Europace.

[9] DeMartino T., Ghoul R.E., Wang L., Bena J., Hazen S.L., Tracy R., Patel S.R., Auckley D., Mehra R. (2016). Oxidative Stress and Inflammation Differentially Elevated in Objective Versus Habitual Subjective Reduced Sleep Duration in Obstructive Sleep Apnea. Sleep.

[10] Peng Y., Yuan G., Overholt J.L., Kumar G.K., Prabhakar N.R. (2003). Systemic and cellular responses to intermittent hypoxia: Evidence for oxidative stress and mitochondrial dysfunction. Adv. Exp. Med. Biol..

[11] Chen L., Zhang J., Gan T.X., Chen-Izu Y., Hasday J.D., Karmazyn M., Balke C.W., Scharf S.M. (2008). Left ventricular dysfunction and associated cellular injury in rats exposed to chronic intermittent hypoxia. J. Appl. Physiol..

[12] Barta K., Szabó Z., Kun C., Munkácsy C., Bene O., Magyar M.T., Csiba L., Lörincz I. (2010). The effect of sleep apnea on QT interval, QT dispersion, and arrhythmias. Clin. Cardiol..

[13] Maeno K., Kasai T., Kasagi S., Kawana F., Ishiwata S., Ohno M., Yamaguchi T., Narui K. (2013). Relationship between atrial conduction delay and obstructive sleep apnea. Heart Vessels.

[14] Voigt L., Haq S.A., Mitre C.A., Lombardo G., Kassotis J. (2011). Effect of obstructive sleep apnea on QT dispersion: A potential mechanism of sudden cardiac death. Cardiology.

[15] Kheirandish-Gozal L., Gozal D. (2019). Obstructive Sleep Apnea and Inflammation: Proof of Concept Based on Two Illustrative Cytokines. Int. J. Mol. Sci..

[16] Saleeb-Mousa J., Nathanael D., Coney A.M., Kalla M., Brain K.L., Holmes A.P. (2023). Mechanisms of Atrial Fibrillation in Obstructive Sleep Apnoea. Cells.

[17] Chen Y.L., Chen Y.C., Wang H.T., Chang Y.T., Fang Y.N., Hsueh S., Liu W.H., Lin P.T., Hsu P.Y., Su M.C. (2022). The Impact of Intermittent Hypoxemia on Left Atrial Remodeling in Patients with Obstructive Sleep Apnea Syndrome. Life.

[18] Zhang L., Hou Y., Po S.S. (2015). Obstructive Sleep Apnoea and Atrial Fibrillation. Arrhythm. Electrophysiol. Rev..

[19] Guilleminault C., Connolly S.J., Winkle R.A. (1983). Cardiac arrhythmia and conduction disturbances during sleep in 400 patients with sleep apnea syndrome. Am. J. Cardiol..

[20] Pépin J.L., Lévy P. (2002). Pathophysiology of cardiovascular risk in sleep apnea syndrome (SAS). Rev. Neurol..

[21] Mansukhani M.P., Kara T., Caples S.M., Somers V.K. (2014). Chemoreflexes, sleep apnea, and sympathetic dysregulation. Curr. Hypertens. Rep..

[22] Narkiewicz K., Somers V.K. (2003). Sympathetic nerve activity in obstructive sleep apnoea. Acta Physiol. Scand..

[23] Maniaci A., Lavalle S., Parisi F.M., Barbanti M., Cocuzza S., Iannella G., Magliulo G., Pace A., Lentini M., Masiello E. (2024). Impact of Obstructive Sleep Apnea and Sympathetic Nervous System on Cardiac Health: A Comprehensive Review. J. Cardiovasc. Dev. Dis..

[24] Mehra R., Benjamin E.J., Shahar E., Gottlieb D.J., Nawabit R., Kirchner H.L., Sahadevan J., Redline S. (2006). Association of nocturnal arrhythmias with sleep-disordered breathing: The Sleep Heart Health Study. Am. J. Respir. Crit. Care Med..

[25] Narkiewicz K., Somers V.K. (1997). The sympathetic nervous system and obstructive sleep apnea: Implications for hypertension. J. Hypertens..

[26] Ghias M., Scherlag B.J., Lu Z., Niu G., Moers A., Jackman W.M., Lazzara R., Po S.S. (2009). The role of ganglionated plexi in apnea-related atrial fibrillation. J. Am. Coll. Cardiol..

[27] Monahan K., Hodges E., Agrawal A., Upender R., Abraham R.L. (2019). Signal-averaged P wave area increases during respiratory events in patients with paroxysmal atrial fibrillation and ob-structive sleep apnea. Sleep Breath.

[28] Lorell B.H., Carabello B.A. (2000). Left ventricular hypertrophy: Pathogenesis, detection, and prognosis. Circulation.

[29] Cloward T.V., Walker J.M., Farney R.J., Anderson J.L. (2003). Left ventricular hypertrophy is a common echocardiographic abnormality in severe obstructive sleep apnea and reverses with nasal continuous positive airway pressure. Chest.

[30] Hedner J., Ejnell H., Caidahl K. (1990). Left ventricular hypertrophy independent of hypertension in patients with obstructive sleep apnoea. J. Hypertens..

[31] Stevenson I.H., Roberts-Thomson K.C., Kistler P.M., Edwards G.A., Spence S., Sanders P., Kalman J.M. (2010). Atrial electrophysiology is altered by acute hypercapnia but not hypoxemia: Implications for promotion of atrial fibrillation in pulmonary disease and sleep apnea. Heart Rhythm.

[32] Gami A.S., Hodge D.O., Herges R.M., Olson E.J., Nykodym J., Kara T., Somers V.K. (2007). Obstructive sleep apnea, obesity, and the risk of incident atrial fibrillation. J. Am. Coll. Cardiol..

[33] Lu Z., Nie L., He B., Yu L., Salim M., Huang B., Cui B., He W., Wu W., Jiang H. (2013). Increase in vulnerability of atrial fibrillation in an acute intermittent hypoxia model: Importance of autonomic imbalance. Auton. Neurosci..

[34] Rossi V.A., Stradling J.R., Kohler M. (2013). Effects of obstructive sleep apnoea on heart rhythm. Eur. Respir. J..

[35] Piccirillo F., Crispino S.P., Buzzelli L., Segreti A., Incalzi R.A., Grigioni F. (2023). A State-of-the-Art Review on Sleep Apnea Syndrome and Heart Failure. Am. J. Cardiol..

[36] Roche F., Gaspoz J.M., Court-Fortune I., Costes F., Geyssant A., Duverney D., Pichot V., Barthélémy J.C. (2003). Alteration of QT rate dependence reflects cardiac autonomic imbalance in patients with obstructive sleep apnea syndrome. Pacing Clin. Electrophysiol..

[37] Camen G., Clarenbach C.F., Stöwhas A.C., Rossi V.A., Sievi N.A., Stradling J.R., Kohler M. (2013). The effects of simulated obstructive apnea and hypopnea on arrhythmic potential in healthy subjects. Eur. J. Appl. Physiol..

[38] Lu D., Wang K. (2025). The relationship between obstructive sleep apnea and atrial remodeling, fibrosis and inflammation. PLoS ONE.

[39] Oliveira W., Campos O., Bezerra Lira-Filho E., Cintra F.D., Vieira M., Ponchirolli A., de Paola A., Tufik S., Poyares D. (2008). Left atrial volume and function in patients with obstructive sleep apnea assessed by real-time three-dimensional echocardiography. J. Am. Soc. Echocardiogr..

[40] Chen Y.L., Chen Y.C., Chang Y.T., Wang H.T., Liu W.H., Chong S.Z., Lin P.T., Hsu P.Y., Su M.C., Lin M.C. (2021). GJA1 Expression and Left Atrial Remodeling in the Incidence of Atrial Fibrillation in Patients with Obstructive Sleep Apnea Syndrome. Biomedicines.

[41] Park J.K., Park J., Uhm J.S., Joung B., Lee M.H., Pak H.N. (2016). Low P-wave amplitude (<0.1 mV) in lead I is associated with displaced inter-atrial conduction and clinical recurrence of paroxysmal atrial fibrillation after radiofrequency catheter ablation. Europace.

[42] Kawano Y., Tamura A., Ono K., Kadota J. (2014). Association between obstructive sleep apnea and premature supraventricular contractions. J. Cardiol..

[43] Horvath C.M., Fisser C., Floras J.S., Sossalla S., Wang S., Tomlinson G., Rankin F., Yatsu S., Ryan C.M., Bradley T.D. (2024). Nocturnal Cardiac Arrhythmias in Heart Failure with Obstructive and Central Sleep Apnea. Chest.

[44] Linz D., Schotten U., Neuberger H.R., Böhm M., Wirth K. (2011). Negative tracheal pressure during obstructive respiratory events promotes atrial fibrillation by vagal activation. Heart Rhythm.

[45] Holtstrand Hjälm H., Fu M., Hansson P.O., Zhong Y., Caidahl K., Mandalenakis Z., Morales D., Ergatoudes C., Rosengren A., Grote L. (2018). Association between left atrial enlargement and obstructive sleep apnea in a general population of 71-year-old men. J. Sleep Res..

[46] Iwasaki Y.K., Kato T., Xiong F., Shi Y.F., Naud P., Maguy A., Mizuno K., Tardif J.C., Comtois P., Nattel S. (2014). Atrial fibrillation promotion with long-term repetitive obstructive sleep apnea in a rat model. J. Am. Coll. Cardiol..

[47] Zipes D.P. (2018). Braunwald’s Heart Disease e-Book: A Textbook of Cardiovascular Medicine.

[48] Bayés de Luna A., Platonov P., Cosio F.G., Cygankiewicz I., Pastore C., Baranowski R., Bayés-Genis A., Guindo J., Viñolas X., Garcia-Niebla J. (2012). Interatrial blocks. A separate entity from left atrial enlargement: A consensus report. J. Electrocardiol..

[49] Chen L.Y., Ribeiro A.L.P., Platonov P.G., Cygankiewicz I., Soliman E.Z., Gorenek B., Ikeda T., Vassilikos V.P., Steinberg J.S., Varma N. (2022). P Wave Parameters and Indices: A Critical Appraisal of Clinical Utility, Challenges, and Future Research-A Consensus Document Endorsed by the International Society of Electrocardiology and the International Society for Holter and Noninvasive Electrocardiology. Circ. Arrhythm. Electrophysiol..

[50] Dilaveris P.E., Gialafos E.J., Andrikopoulos G.K., Richter D.J., Papanikolaou V., Poralis K., Gialafos J.E. (2000). Clinical and electrocardiographic predictors of recurrent atrial fibrillation. Pacing Clin. Electrophysiol..

[51] Dilaveris P.E., Gialafos E.J., Sideris S.K., Theopistou A.M., Andrikopoulos G.K., Kyriakidis M., Gialafos J.E., Toutouzas P.K. (1998). Simple electrocardiographic markers for the prediction of paroxysmal idiopathic atrial fibrillation. Am. Heart J..

[52] Marks D., Ho R., Then R., Weinstock J.L., Teklemariam E., Kakadia B., Collins J., Andriulli J., Hunter K., Ortman M. (2021). Real-world experience with implantable loop recorder monitoring to detect subclinical atrial fibrillation in patients with cryptogenic stroke: The value of p wave dispersion in predicting arrhythmia occurrence. Int. J. Cardiol..

[53] Zeng C., Wei T., Zhao R., Wang C., Chen L., Wang L. (2003). Electrocardiographic diagnosis of left atrial enlargement in patients with mitral stenosis: The value of the P-wave area. Acta Cardiol..

[54] Morris J.J., Estes E.H., Whalen R.E., Thompson H.K., McIntosh H.D. (1964). P-Wave Analysis in Valvular Heart Disease. Circulation.

[55] Tanaka N., Okada M., Tanaka K., Harada S., Kawahira M., Hirao Y., Onishi T., Koyama Y., Fujii K., Watanabe H. (2022). Untreated sleep apnea and left atrial dilatation in patients with atrial fibrillation prior to catheter ablation. Eur. Heart J..

[56] Jazi M.H., Amra B., Yazdchi M.R., Jahangiri M., Tabesh F., Gholamrezaei A. (2014). P wave duration and dispersion in Holter electrocardiography of patients with obstructive sleep apnea. Sleep Breath..

[57] Qureshi W.T., Nasir U.B., Alqalyoobi S., O’Neal W.T., Mawri S., Sabbagh S., Soliman E.Z., Al-Mallah M.H. (2015). Meta-Analysis of Continuous Positive Airway Pressure as a Therapy of Atrial Fibrillation in Obstructive Sleep Apnea. Am. J. Cardiol..

[58] Labarca G., Dreyse J., Drake L., Jorquera J., Barbe F. (2020). Efficacy of continuous positive airway pressure (CPAP) in the prevention of cardiovascular events in patients with obstructive sleep apnea: Systematic review and meta-analysis. Sleep Med. Rev..

[59] Abuzaid A.S., Al Ashry H.S., Elbadawi A., Ld H., Saad M., Elgendy I.Y., Elgendy A., Mahmoud A.N., Mentias A., Barakat A. (2017). Meta-Analysis of Cardiovascular Outcomes with Continuous Positive Airway Pressure Therapy in Patients with Obstructive Sleep Apnea. Am. J. Cardiol..

[60] Yu J., Zhou Z., McEvoy R.D., Anderson C.S., Rodgers A., Perkovic V., Neal B. (2017). Association of Positive Airway Pressure with Cardiovascular Events and Death in Adults with Sleep Apnea: A Systematic Review and Meta-analysis. JAMA.

[61] Hnatkova K., Andršová I., Toman O., Smetana P., Huster K.M., Šišáková M., Barthel P., Novotný T., Schmidt G., Malik M. (2021). Spatial distribution of physiologic 12-lead QRS complex. Sci. Rep..

[62] Chundusu C.M., David N., Badung D. (2020). Normal P, QRS & T Axis on an Electrocardiogram (ECG) as Seen in Plateau Specialist Hospital, Jos. Central Nigeria. Online J. Cardiol. Res. Rep..

[63] Zipes D.P., Rubart M. (2006). Neural modulation of cardiac arrhythmias and sudden cardiac death. Heart Rhythm.

[64] Davies R.A., Ladouceur V.B., Green M.S., Joza J., Juurlink D.N., Krahn A.D., McMurtry M.S., Roberts J.D., Roston T.M., Sanatani S. (2023). The 2023 Canadian Cardiovascular Society Clinical Practice Update on Management of the Patient with a Prolonged QT Interval. Can. J. Cardiol..

[65] Naruse Y., Tada H., Satoh M., Yanagihara M., Tsuneoka H., Hirata Y., Ito Y., Kuroki K., Machino T., Yamasaki H. (2013). Concomitant obstructive sleep apnea increases the recurrence of atrial fibrillation following radiofrequency catheter ablation of atrial fibrillation: Clinical impact of continuous positive airway pressure therapy. Heart Rhythm.

[66] Metwally M., Roshdy S., Ghany M.A., Abd El Razik A. (2014). P wave dispersion and severity of obstructive sleep apnea syndrome. Egypt. J. Chest Dis. Tuberc..

[67] Karamanlı H., Aygün F., Akgedik R. (2016). Relationship Between Severity of Obstructive Sleep Apnea and PR Interval. Koşuyolu Heart J..

[68] Koehler U., Fus E., Grimm W., Pankow W., Schäfer H., Stammnitz A., Peter J.H. (1998). Heart block in patients with obstructive sleep apnoea: Pathogenetic factors and effects of treatment. Eur. Respir. J..

[69] Kawana F., Kasai T., Maeno K., Momomura S., Narui K. (2008). Atrioventricular block during the phasic events of REM sleep in a patient with severe obstructive sleep apnea syndrome. J. Clin. Sleep Med..

[70] Maeno K., Kasai A., Setsuda M., Nishiyama A., Sakabe S., Ohnishi T., Saito K., Nishikawa H. (2009). Advanced atrioventricular block induced by obstructive sleep apnea before oxygen desaturation. Heart Vessels.

[71] Geovanini G.R., Lorenzi-Filho G. (2018). Cardiac rhythm disorders in obstructive sleep apnea. J. Thorac. Dis..

[72] May A.M., Van Wagoner D.R., Mehra R. (2017). OSA and Cardiac Arrhythmogenesis: Mechanistic Insights. Chest.

[73] Baranchuk A. (2012). Sleep apnea, cardiac arrhythmias, and conduction disorders. J. Electrocardiol..

[74] Patel N., Donahue C., Shenoy A., Patel A., El-Sherif N. (2017). Obstructive sleep apnea and arrhythmia: A systemic review. Int. J. Cardiol..

[75] Quintal J., Parreira L., Esteves A.F., Candjondjo A.P., Ferreira J.S., Coelho R.A., Duarte P., Sousa S., Rijo C., Amador P. (2023). Severity of obstructive sleep apnea is associated with the presence of frequent premature ventricular contractions. EP Eur..

[76] Van Gelder I.C., Rienstra M., Bunting K.V., Casado-Arroyo R., Caso V., Crijns H., De Potter T.J.R., Dwight J., Guasti L., Hanke T. (2024). 2024 ESC Guidelines for the management of atrial fibrillation developed in collaboration with the European Association for Cardio-Thoracic Surgery (EACTS). Eur. Heart J..

[77] Kadhim K., Middeldorp M.E., Elliott A.D., Agbaedeng T., Gallagher C., Malik V., Wong C.X., McEvoy R.D., Kalman J.M., Lau D.H. (2021). Prevalence and Assessment of Sleep-Disordered Breathing in Patients with Atrial Fibrillation: A Systematic Review and Meta-analysis. Can. J. Cardiol..

[78] Moula A.I., Parrini I., Tetta C., Lucà F., Parise G., Rao C.M., Mauro E., Parise O., Matteucci F., Gulizia M.M. (2022). Obstructive Sleep Apnea and Atrial Fibrillation. J. Clin. Med..

[79] Kadhim K., Middeldorp M.E., Elliott A.D., Jones D., Hendriks J.M.L., Gallagher C., Arzt M., McEvoy R.D., Antic N.A., Mahajan R. (2019). Self-Reported Daytime Sleepiness and Sleep-Disordered Breathing in Patients with Atrial Fibrillation: SNOozE-AF. Can. J. Cardiol..

[80] Traaen G.M., Øverland B., Aakerøy L., Hunt T.E., Bendz C., Sande L., Aakhus S., Zaré H., Steinshamn S., Anfinsen O.G. (2020). Prevalence, risk factors, and type of sleep apnea in patients with paroxysmal atrial fibrillation. Int. J. Cardiol. Heart Vasc..

[81] Kapur V.K., Auckley D.H., Chowdhuri S., Kuhlmann D.C., Mehra R., Ramar K., Harrod C.G. (2017). Clinical Practice Guideline for Diagnostic Testing for Adult Obstructive Sleep Apnea: An American Academy of Sleep Medicine Clinical Practice Guideline. J. Clin. Sleep Med..

[82] Kanagala R., Murali N.S., Friedman P.A., Ammash N.M., Gersh B.J., Ballman K.V., Shamsuzzaman A.S., Somers V.K. (2003). Obstructive sleep apnea and the recurrence of atrial fibrillation. Circulation.

[83] Holmqvist F., Guan N., Zhu Z., Kowey P.R., Allen L.A., Fonarow G.C., Hylek E.M., Mahaffey K.W., Freeman J.V., Chang P. (2015). Impact of obstructive sleep apnea and continuous positive airway pressure therapy on outcomes in patients with atrial fibrillation-Results from the Outcomes Registry for Better Informed Treatment of Atrial Fibrillation (ORBIT-AF). Am. Heart J..

[84] Fein A.S., Shvilkin A., Shah D., Haffajee C.I., Das S., Kumar K., Kramer D.B., Zimetbaum P.J., Buxton A.E., Josephson M.E. (2013). Treatment of obstructive sleep apnea reduces the risk of atrial fibrillation recurrence after catheter ablation. J. Am. Coll. Cardiol..

[85] Li L., Wang Z.W., Li J., Ge X., Guo L.Z., Wang Y., Guo W.H., Jiang C.X., Ma C.S. (2014). Efficacy of catheter ablation of atrial fibrillation in patients with obstructive sleep apnoea with and without continuous positive airway pressure treatment: A meta-analysis of observational studies. Europace.

[86] McEvoy R.D., Antic N.A., Heeley E., Luo Y., Ou Q., Zhang X., Mediano O., Chen R., Drager L.F., Liu Z. (2016). CPAP for Prevention of Cardiovascular Events in Obstructive Sleep Apnea. N. Engl. J. Med..

[87] Yılmaz M., Kayançiçek H., Gözel N., Bilen M.N., Kurtoğlu E., Seçen Ö., Öner P., Demirkıran S., Uku Ö., Çekici Y. (2020). Spotlights on some electrocardiographic paradigms: How should we evaluate normal reference values of Tp-Te interval, Tp-Te dispersion and Tp-Te/QT ratio?. Adv. Clin. Exp. Med..

[88] Gupta P., Patel C., Patel H., Narayanaswamy S., Malhotra B., Green J.T., Yan G.X. (2008). T(p-e)/QT ratio as an index of arrhythmogenesis. J. Electrocardiol..

[89] Bacharova L., Triantafyllou E., Vazaios C., Tomeckova I., Paranicova I., Tkacova R. (2015). The effect of obstructive sleep apnea on QRS complex morphology. J. Electrocardiol..

[90] Gupta S., Cepeda-Valery B., Romero-Corral A., Shamsuzzaman A., Somers V.K., Pressman G.S. (2012). Association between QRS duration and obstructive sleep apnea. J. Clin. Sleep Med..

[91] Marinheiro R., Parreira L., Amador P., Mesquita D., Farinha J., Fonseca M., Duarte T., Lopes C., Fernandes A., Caria R. (2019). Ventricular Arrhythmias in Patients with Obstructive Sleep Apnea. Curr. Cardiol. Rev..

[92] Smith J.H., Baumert M., Nalivaiko E., McEvoy R.D., Catcheside P.G. (2009). Arousal in obstructive sleep apnoea patients is associated with ECG RR and QT interval shortening and PR interval lengthening. J. Sleep Res..

[93] Mehra R., Chung M.K., Olshansky B., Dobrev D., Jackson C.L., Kundel V., Linz D., Redeker N.S., Redline S., Sanders P. (2022). Sleep-Disordered Breathing and Cardiac Arrhythmias in Adults: Mechanistic Insights and Clinical Implications: A Scientific Statement From the American Heart Association. Circulation.

[94] Namtvedt S.K., Randby A., Einvik G., Hrubos-Strøm H., Somers V.K., Røsjø H., Omland T. (2011). Cardiac arrhythmias in obstructive sleep apnea (from the Akershus Sleep Apnea Project). Am. J. Cardiol..

[95] He H., Lim V.G., Weight N., Lachlan T., Osman F. (2025). Incident Arrhythmias Detected Using Implantable Loop Recorders in Obstructive Sleep Apnoea. Rev. Cardiovasc. Med..

[96] Laczay B., Faulx M.D. (2021). Obstructive Sleep Apnea and Cardiac Arrhythmias: A Contemporary Review. J. Clin. Med..

[97] Shahrbabaki S.S., Mittinty M., Tonchev I.R., Strong C., Chapman D., Lorensini S., Jenkins E., Catcheside P., Adams R., Eckert D.J. (2025). Continuous Positive Airway Pressure Reduces Nocturnal Arrhythmia Avalanche Burden in Obstructive Sleep Apnea: An Adherence-Dependent Effect. Heart Rhythm.

[98] Paul T., Hassan O., McCrae C.S., Islam S.K., Mosa A.S.M. (2025). An Explainable Fusion of ECG and SpO_2_-Based Models for Real-Time Sleep Apnea Detection. Bioengineering.

[99] Shahrbabaki S.S., Strong C., Chapman D., Tonchev I., Jenkins E., Lechat B., Nguyen D.P., Mittinty M., Catcheside P., Eckert D.J. (2025). Characterisation of nocturnal arrhythmia avalanche dynamics: Insights from generalised linear model analysis. J. Sleep Res..

[100] Sheta A., Turabieh H., Thaher T., Too J., Mafarja M., Hossain M.S., Surani S.R. (2021). Diagnosis of Obstructive Sleep Apnea from ECG Signals Using Machine Learning and Deep Learning Classifiers. Appl. Sci..

[101] Haghighat S., Joghatayi M., Issa J., Azimian S., Brinz J., Ashkan A., Chaurasia A., Rahimian Z., Sangalli L. (2025). Diagnostic accuracy of artificial intelligence for obstructive sleep apnea detection: A systematic review. BMC Med. Inform. Decis. Mak..

[102] Abd-Alrazaq A., Aslam H., AlSaad R., Alsahli M., Ahmed A., Damseh R., Aziz S., Sheikh J. (2024). Detection of Sleep Apnea Using Wearable AI: Systematic Review and Meta-Analysis. J. Med. Internet Res..

[103] Baty F., Boesch M., Widmer S., Annaheim S., Fontana P., Camenzind M., Rossi R.M., Schoch O.D., Brutsche M.H. (2020). Classification of Sleep Apnea Severity by Electrocardiogram Monitoring Using a Novel Wearable Device. Sensors.

[104] Bsoul M., Minn H., Tamil L. (2011). Apnea MedAssist: Real-time sleep apnea monitor using single-lead ECG. IEEE Trans. Inf. Technol. Biomed..

[105] Faust O., Acharya U.R., Ng E., Fujita H. (2016). A review of ECG-based diagnosis support systems for obstructive sleep apnea. J. Mech. Med. Biol..

